# Essentials of Genitourinary and Venereal Diseases

**Published:** 1906-09

**Authors:** 


**Essentials of Genitourinary and Venereal Diseases.** By **Starling S. Wilcox**, M.D., Professor of Genitourinary Diseases and Syphilology, Starling Medical College, Columbus, Ohio. 12mo. of 313 pages, illustrated. Philadelphia and London: W. B. Saunders Company. 1906. (Cloth, $1.00 net).

It is a difficult matter to crowd into a compend all the material necessary to an understanding of so complicated a subject as genitourinary work, to say nothing of venereal diseases; yet Wilcox has made a brave effort to combine the two subjects in one small volume of the Saunders series of compends and has succeeded in presenting a book which is, at least, helpful to a student in a broad sense, for it gives him an idea of the depth of the subject and its manifold difficulties. One cannot very well approve of the subcutaneous ligation in varicocele, nor of castration for prostatic hypertrophy, any more than one can accept the suggestion to irrigate the urethra by means of catheter.

The treatment of gonorrhea by irrigation is given its proper place in the forefront of other methods of local procedures, and did some of the insurgents of the profession use the care in irrigating by the Valentine method which Wilcox so strongly advises, they would get better results; also be less likely to beat the tom-tom of ridicule, and hold up their own mistakes as routine results of a most excellent treatment. Wilcox's command of the subject is comprehensive and his manner of presenting the details of symptomatology and treatment is admirable. The book cannot help being of great service to the student; and it might be studied to great advantage by many a general practitioner whose tendency is to make light of the venereal diseases, and whose lack of detailed knowledge in the genitourinary field leads him to practise in this branch of medicine with a bottle of acid and another of alkaline cystitis tablets. N. W. W.

